# Effect of feed enriched by products formulated from coconut water, palm sap sugar, and mushroom on the chemical composition of feed and carcass, growth performance, body indices, and gut micromorphology of giant gourami, *Osphronemus goramy* (Lacepède, 1801), juveniles

**DOI:** 10.12688/f1000research.124706.2

**Published:** 2023-09-11

**Authors:** Azrita Azrita, Hafrijal Syandri, Netti Aryani, Ainul Mardiah

**Affiliations:** 1 Department of Aquaculture, Faculty of Fisheries and Marine Science, Universitas Bung Hatta, Padang, West Sumatera, 25113, Indonesia; 2 Department of Aquaculture, Faculty of Fisheries and Marine Science, Universitas Riau, Pekanbaru, 28293, Indonesia; 3 Department of Aquaculture, Faculty of Fisheries and Marine Science, Universitas Nahdlatul Ulama, Padang, West Sumatera, 25118, Indonesia

**Keywords:** Giant gourami, amino acid profile, growth performance, palm sap sugar, coconut water, gut micromorphology

## Abstract

**Background:** Giant gourami, *Osphronemus goramy* is the most important freshwater fish species produced by aquaculture in Indonesia. This study aimed to the effects of various newly formulated products on the amino acid composition of the diet and whole-body carcass, and to analyse the growth coefficient, body indices, and gut micromorphology.

**Methods:** One hundred gram of palm sap sugar was cooked in 1.1 litre of fresh water for fifteen minutes, to create 1 litre of 11% palm sap sugar solution (after some of it had been boiled off). Two litres of coconut water were then mixed with the litre of palm sugar solution. One litre of this product was added in turn to 2 g of *Aspergillus niger* (CP2), 2 g of *Rhizopus oligosporus* (CP3), and 2 g of *Saccharomyces cerevisiae* (CP4), while freshwater was used as a control (labeled CP1). Aquafeed was added to CP1, CP2, CP3, and CP4, to make diets labeled KP1, KP2, KP3, and KP4. The dosage was 150 ml/kg of feed. Juvenile giant gourami (initial weight 50±0.25 g and length 13.2±0.07 cm) were reared in triplicate net frames (2×1×1 m; water volume 1.5 m ^3^) in a freshwater concrete pond with a stocking density of 30 juveniles/net.

**Results: **The results supported our hypothesis that different product formulations have a significant effect (P < 0.05) on aquafeed nutrition and the whole-body carcass, growth coefficient, feed utilization, body indices, and gut micromorphology of giant gourami juveniles. The thermal growth coefficient strongly correlated with the daily growth coefficient (r ^2^ = 92%), condition factor (r ^2^ = 77%), protein efficiency ratio (r ^2 ^= 75%), while a moderate relationship with the feed intake ( r ^2^ = 69%).

**Conclusions: **Diet KP3 contains higher total amino acids in diets and carcasses and leads to feed efficiency and better growth for giant gourami.

## Introduction

In this decade, the production of capture fisheries has decreased; meanwhile, the demand for fish products for human consumption is increasing. Therefore, according to the Food and Agriculture Organisation, 60% of fisheries production in the future will come from aquaculture activities and this figure will continue to rise. ^1^ The utilization of a variety of fish for aquaculture has now increased the need for commercial feed. ^2^^–^^5^ At the same time, for aquaculture operations, the cost of aquafeed is still a significant challenge. ^2^^,^^6^^–^^8^ On the other hand, commercial feed produced by factories still does not contain complete nutrition for fish growth, while being acknowledged for its positive effects on food safety. ^9^^–^^11^ In this context, enriching fish feed with cost-effective natural ingredient resources is key to increasing feed nutrient quality and feed efficiency in commercial fish farming and ensuring the sustainability of aquaculture operations. ^2^^,^^12^^,^^13^

The target is fish feed that is wealthy in many important nutrients, including protein, fat, vitamins, and minerals that cultured fish can utilize to increase their growth rate and survival and that is beneficial for human health. ^4^^,^^14^^–^^16^ Therefore, novel approaches have been developed by scientists to improve the nutrition of fish feeds, such as feed supplemented with EPA and DHA, ^17^ iodine and selenium, ^10^ methionine, ^18^ fish oil, ^11^^,^^19^ and soybean oil. ^20^ In addition, supplementing probiotics into the diet ^21^ and supplemental glycine, prebiotics, and nucleotides in a soybean meal-based diet have been studied. ^22^

The progress of aquaculture biotechnology has stimulated the interest of scientists in improving aquatic animal production, for example, to increase giant gourami production. One of the experimental techniques is to increase feed nutrition used for this purpose, such as, the use of fish meal and Azolla ( *Azolla pinnata*) flour as a feed ingredient for giant gourami, ^23^ and the utilization of new products formulated from water coconut, palm sap sugar, and fungus for the enrichment of commercial feed. ^9^ Additional research has involved a diet supplemented using glutamine, ^24^ feed supplemented with a growth hormone, ^25^ and substitute fish meal incorporating chicken feather. ^26^ Whether using coconut water and palm sap sugar fermented with mushrooms ( *Aspergillus niger, Rhizopus oligosporus, and Saccharomyces cerevisiae*) affects the amino acid composition of the diet, body carcass, growth coefficient, and body indices is still not understood.

Coconut water has extraordinary nutritional value and contains supplements for health like minerals, amino acids, fatty acids, vitamins, enzymes, organic acids, and several phenolic compositions. ^27^^–^^30^ Palm sap sugar also has health benefits due to its essential nutrient content, such as a low glycaemic index, and it contains antioxidants, vitamins, and minerals. ^31^^–^^34^ Meanwhile, mushrooms have been widely used in fermentation due to their ability to degrade antigenic proteins in fish feed ingredients. ^7^^,^^35^^,^^36^ Additionally, coconut water is a functional food that can protect the lens from diabetic cataract development in rats. ^37^ Coconut water is also a treatment for burning pain during urination, dysuria, gastritis, increasing semen, and indigestion. ^38^

On the other hand, Azrita *et al.* ^9^ have reported using new formulations of products containing coconut water and palm sap sugar that are fermented with various mushrooms involving a dosage of 300 ml/kg feed. Their newly formulated products can increase fatty acid levels in the diet and whole-body carcasses. Besides that, they also improve giant gourami's growth performance and feed efficiency.

However, the effect of these new formulation products at a dosage of 150 ml/kg feed on the diet amino acid composition, and body meat's amino acid composition has not yet been analyzed. In line with that, the relationships between thermal growth coefficient and condition factor, daily growth coefficient, and feed utilization coefficient, including body indices parameters, as well as the gut micromorphology of giant gourami, have not yet been analyzed.

We hypothesized that commercial aquafeed combined with different newly formulated products at the dosage of 150 ml/kg feed could improve the amino acids compositions of the aquafeed and whole-body carcass, body indices, and gut micromorphology. Hence, this investigation's first purpose was to analyze the effect of various newly formulated products on the diet's proximate compositions, amino acid composition, and whole-body carcass. The second aim was to analyze the impact of newly formulated products on condition factor, the growth coefficient and relation to thermal growth coefficient, body indices, and gut micromorphology in giant gourami juveniles.

## Methods

### Ethical approval

The Research and Community Service Ethics Committee at Universitas Bung Hatta, West Sumatera, Indonesia approved this research (89/LPPM/Hatta/III-2022) which followed the ARRIVE guidelines. The Ministry of Education, Culture, Research and Technology of the Republic of Indonesia funded the research under grant No. 076/E5/PG.02.00 PT/2022 on March 16, 2022. Approval was given by the ethics committee to collect and rear juvenile of giant gourami in the aquaculture laboratory, Faculty of Fisheries and Marine Science at Universitas Bung Hatta. All efforts were made to relieve the suffering of experimental animals. Therefore, the animal did not suffer for this study, and they were still in good condition when returned to the pond after research was completed. Where some fish were euthanized, this was carried out by piercing part of the fish’s brain. Giant gourami are not classified as a protected animal according to Indonesian legislation.

### Preparation of formulated product

We prepared 100 g of palm sap sugar by traditional production and cooked it in 1.1 litre of fresh water for fifteen minutes at 60 ^0^ C, and only got 1 litre of palm sap sugar solution due to some of it being boiled off. Then, this was cooled in an open space for twenty minutes. Furthermore, we also prepared 2.0 litres of mature coconut water ( *Cocos nucifera* L *.*) and mixed it with the 1.0 litre of palm sap sugar solution. The product was stored for ten minutes in a cool air-conditioned room. A total of 3.0 litres of the formulated product was divided into three parts of 1.0 litre each. We added 2 g of *Aspergillus niger* (labeled as CP2 product) to the first part of the formulated product solution, 2 g of *Rhizopus oligosporus* (labeled as CP3 product) to the second part, and 2 g of *Saccharomyces cerevisiae* (labeled as CP4 product) to the third portion. The CP2, CP3, and CP4 products were fermented for 48 hrs in a jerry can (2.0 litres) using an Aerasi PUJIMAC, MAC-40 K 40 L/min. The products of CP2, CP3, and CP4 were used to enrich the nutrition of commercial aquafeed (781-2, PT. Japfa Comfeed Indonesia, Tbk) and labeled as the KP2, KP3, and KP4 diets. The aquafeed was supplemented with freshwater (labeled as the KP1 diet; placebo).

### Preparation of experimental diets

Giant gourami juveniles were adapted for one month to standard feed, namely floating commercial aquafeed 781-2 (pellet size 2 mm), which contained 10.66% water content, 30.10% crude protein, 4.09% crude fat, 45.35% total carbohydrates, 2.5% ash, and 9.18% crude fibre. The minerals in the commercial feed were 280.08 mg/100 g Na, 1415.02 mg/100 g Ca, 1358.07 mg/100 g K, 1200.31 mg/100 g P, 292.03 mg/100 g Mg, 18.14 mg/100 g Fe, and 13.83 mg/100 g Zn. The aquafeed was added to freshwater to create the KP1 diet as observed, and the formulated CP2, CP3, and CP4 products were added to the aquafeed at a dosage of 150 ml/kg of feed to create the enriched fish diets. The formulated product added to the aquafeed was mixed manually with it for three minutes to obtain maximum homogenization and then the blend was dried in the open air for thirty minutes. Thereafter, it was given to the trial animal.

### Experimental procedures and sampling

In the present study, we measured fish weight using AD-600i scales with 0.001 g accuracy (ACIS model number AD-600i, China). At the same time, a meter ruler with 1 mm accuracy was used to estimate the body length. A total of 360 sago strain juveniles of giant gourami were counted; the initial mean weight was 50 ± 0.25 g, and the initial length was 13.2±0.07 cm. For rearing juveniles, twelve nets framed with 2 m ^3^ (2 × 1 × 1 m) PVC pipe (water volume of 1.5 m ^3^) were placed inside two freshwater concrete ponds with a size of 18 m ^3^ (6 × 2 × 1.5 m). This experiment consisted of four treatments and three replications, and each frame net was stocked with 30 juveniles. The giant gourami were fed the KP1, KP2, KP3, and KP4 diets three times a day (08:00, 12:00, and 17:00 hrs) during the 90-day feeding trial. Juveniles of giant gourami were fed at a 3% body weight rate per day based on the percentage of stored biomass. Fish samples were collected every 30 days for body weight and length measurements. Ten fish per net frame were collected and anesthetized orally using clove oil. Then, their lengths and weights were measured. Prior to sampling, the fish fasted for 24 hrs to empty their intestinal contents.

### Proximate and amino acid composition

The diet samples and proximate carcass composition were analyzed using standard AOAC methods. ^39^ The matter was dried to a constant weight at 105°C. We used the standard Kjeldahl method to analyse crude protein (N × 6.25). We used the Soxhlet method with ether extraction to analyse crude lipids; the ash was incinerated at 550°C for 16 hrs, whereas gross energy was measured in a bomb calorimeter. The amino acid composition was determined by using a high-performance liquid chromatography (HPLC) system consisting of a water 1525 binary HPLC pump, 717 autosamplers (water ^®^), and water 2475 multi λ fluorescence detector optics (wavelengths: 250 nm for excitation and 395 nm for emission). It was hydrolysed in triplicate with 6 N hydrochloric acid for 24 hrs at 11°C. ^40^

### Nutrient utilization and body indices

The growth coefficients in the fish experiments were measured by using the thermal growth coefficient (TGC), daily growth coefficient (DGC), total feed intake (FI), and protein efficiency ratio (PER) of giant gourami, assessed using the following formulae:

$$\mathrm{TGC}=\left[\left(\text{final weight}\;\left(g\right)\right)⅓–\left(\text{initial weight}\;\left(g\right)\right)⅓\right]/[\left(\text{mean water temperature}\;\left({}^{\circ}C\right)\right)\times \text{duration of rearing period}\;\left(\mathrm{day}\right)]\times 1000$$

$$\mathrm{DGC}=\left(\mathrm{Wf}⅓–\mathrm{Wi}⅓\right)/\text{duration of rearing period}\;\left(\mathrm{day}\right)\times 100$$

$$\mathrm{FI}\;\mathrm{as}\;\text{feed}\;\left(\mathrm{FI}\;\mathrm{as}\;\text{feed in}\;g/\text{fish}/\mathrm{day}\right)=\text{Total feed}\;\mathrm{fed}/\left(n\times t\right)$$

PER=wetweight gain/total protein intake

Three fish from each net frame were sacrificed and dissected immediately to determine the Condition factor (CF), Viscerosomatic index (GSI%), Hepatosomatic index (HSI%), Visceral fat-somatic indexes (VFSI%), and Bilesomatic index (BSI) as given below:

$$\mathrm{CF}=100\times \left[\text{weight of the juvenile}\;\left(g\right)/\text{Length of juvenile}\;\left({\mathrm{cm}}^{3}\right)\right]$$

$$\mathrm{GSI}=100\times \left[\text{viscera weight}\;\left(g\right)/\text{whole body weight}\;\left(g\right)\right]$$

$$\mathrm{HSI}=100\times \left[\text{liver weight}\;\left(g\right)/\text{whole body weight}\;\left(g\right)\right]$$

$$\text{VFSI}=100\times \left[\text{visceral}\;\mathrm{fat}\;\text{weight}\;\left(g\right)/\text{whole body weight}\;\left(g\right)\right]$$

$$\mathrm{BSI}=100\times \left[\text{Bile weight}\;\left(g\right)/\text{weight of liver}\;\left(g\right)\right]$$

### Histological examination of the gut

**Figure 1.**
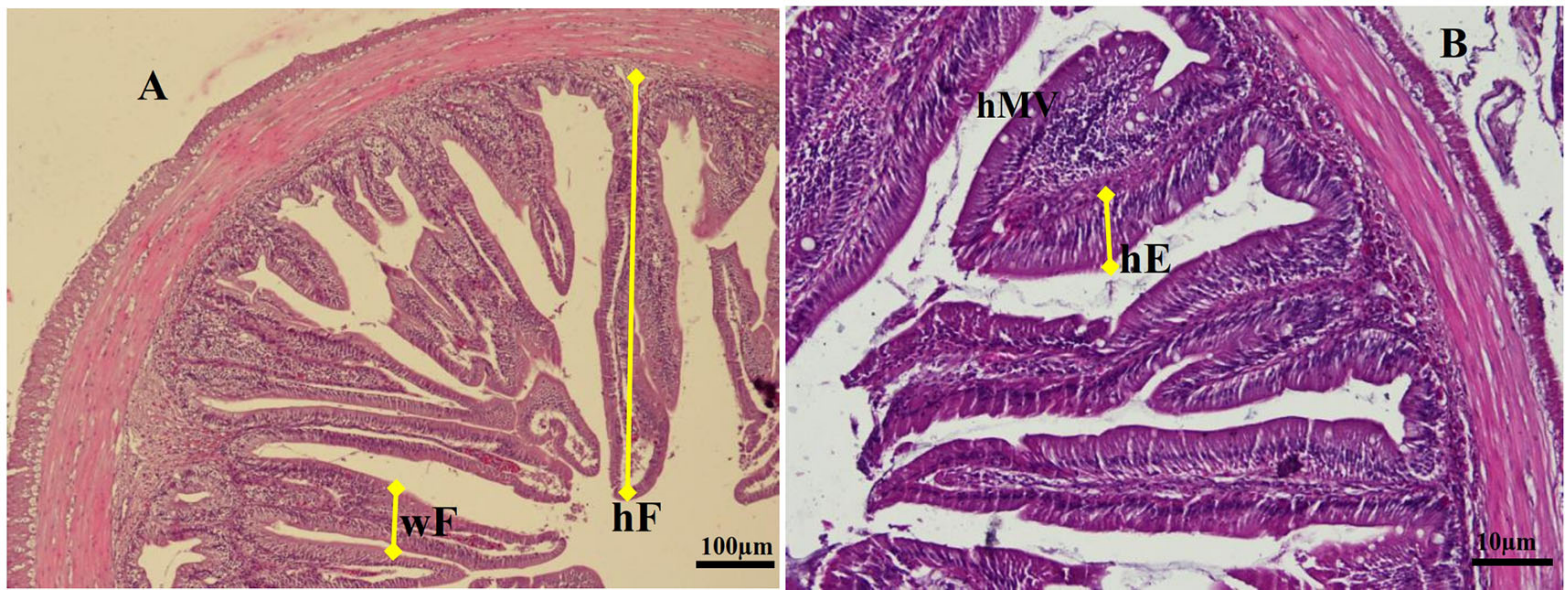
Transversal section photomicrographs of giant gourami juvenile foregut. (A) Fold height and fold width were analyzed in a lower magnification of objective lens of microscope (magnification × 100), (B) Enterocytes height and microvilli height were analyzed using a higher magnification of an objective lens microscope (magnification × 200). hF = fold height, wF = fold width, hE = enterocyte height, hMV = microvillus height (hematoxylin and eosin).

For histological analyses, each gut specimen of the animal was cut into the foregut, midgut, and hindgut. Moreover, the cells were cleaned in saline solution and fixed in Bouin's fixative solution for 24 hrs. After sequential dehydration steps in alcohol, the gut samples were embedded in paraffin. The implanted tissue blocks were sectioned at 5 μm, and sections were consistently stained with Haematoxylin-eosin and observed under a light microscope (Olympus IX71) equipped with Image-Pro Plus 7.0 software, Japan. The digitalized analysis measures the micrometer length of various enteric structures of gut images. We determined the average fold height (hF), fold width (wF), and enterocyte height (hMV) of the gut per slice (5 fields per individual sample) according to procedures described by Li *et al.* ^18^ The specific measurement method of gut samples is shown in Figure 1.

### Pond water quality

The water quality values of the freshwater concrete ponds that were used to rear the giant gourami juveniles were recorded weekly. The water samples were collected at 10:00 am at a depth of 20 cm from each concrete pond to determine the water temperature, dissolved oxygen, and pH value. In addition, we also measured the total alkalinity, hardness, and nitrates of the water in the pond experiments. A thermometer (Celsius scale) was used to measure water temperature. To measure water dissolved oxygen (O _2_; mg L ^-1^), we used an oxygen meter (YSI Model 52, Yellow Instrument Co, Yellow Spring, OH USA). A digital pH meter (Mini 0–14 pH IQ, Scientific Cemo Science, Thailand) was used to determine the pH values of water in the experiments. The level of nitrate-nitrogen (NO _3_-N; mg L ^-1^), alkalinity (mg L ^-1^), and hardness (mg L ^-1^) were measured according to standard procedures. ^41^

### Calculations and statistical method

The data from this study were reported in the form of the mean ± standard deviation for each treatment. Data were analysed using the SPSS 16.0 software package (SPSS; Chicago, IL). Normality was tested using the Kolmogorov–Smirnov statistic. Homogeneity was checked using absolute residuals according to Levine's test. One-way ANOVA was used to determine the treatment effect, followed by a post-hoc Duncan's multiple range test. ^42^ To create the figures, Microsoft Office Professional Plus 2019 was used.

## Results

### Proximate and amino acid profiles of the diets

Commercial feed supplemented with different formulated products with the dosage of 150 ml/kg of feed significantly affects the proximate composition of diets. One-way ANOVA results showed a marginal interaction among treatments in the case of protein content (F _(3,8)_ = 1.522, *P* = 0.282), fat (F _(3,8)_ = 5.663, *P* = 0.022), carbohydrates (F _(3,8)_ = 1.862, *P* = 0.214), crude fibre (F _(3,8)_ = 1.445, *P* = 0.300), and ash (F _(3,8)_ = 0.272, *P* = 0.844), and the total energy content (F _(3,8)_ =1.112, *P* = 0.400) differed considerably ( *P* < 0.05) among the four diets ( Table 1). Duncan's Post-hoc test revealed that the protein content (21.6967 ± 0.17%) was significantly higher ( *P* < 0.05) in the KP3 diet than in the other treatments, while the carbohydrate (31.19 ± 0.38%), crude fibre (2.82 ± 0.06%), and ash (6.57 ± 0.04%) contents were significantly higher ( *P* < 0.05) in the KP3 diet than in the other diets. Conversely, the total energy content was 240.88 ± 0.74 (kg calories/100 g), which was significantly higher ( *P* < 0.05) in the KP3 diets than in the KP1, KP2, and KP4 diets ( Table 1).

**Table 1.** The experimental diets' proximate and amino acid composition (% dry matter). Mean ± SD *.

	KP1	KP2	KP3	KP4
	%, dry weight basis			
*Proximate composition*				
Dry matter	38.42 ± 0.25 ^a^	38.27 ± 0.01 ^a^	37.59 ± 0.16 ^a^	38.41± 0.10 ^a^
Crude protein	19.68 ± 0.41 ^a^	20.27 ± 0.13 ^b^	21.70 ± 0.18 ^c^	20.44 ± 0.10 ^d^
Crude lipid	3.41 ± 0.02 ^a^	3.67 ± 0.13 ^b^	3.50 ± 0.02 ^ac^	3.48 ± 0.04 ^ad^
Carbohydrate	26.37 ± 0.17 ^a^	29.50 ± 0.54 ^b^	31.19 ± 0.38 ^c^	30.57 ± 0.06 ^d^
Crude fibre	2.23 ± 0.05 ^a^	2.36 ± 0.01 ^b^	2.82 ± 0.06 ^c^	2.45 ± 0.06 ^d^
Ash	2.75 ± 0.03 ^a^	6.66 ± 0.05 ^b^	6.57 ± 0.04 ^c^	6.67 ± 0.06 ^d^
Energy total (kg calorie/100 g)	240.87 ± 0.38 ^a^	234.41 ± 0.30 ^b^	240.88 ± 0.74 ^ac^	237.11 ± 0.43 ^d^
*Amino acid composition*				
EAA				
Leucine	1.36 ± 0.01 ^a^	1.42 ± 0.01 ^b^	1.46 ± 0.01 ^c^	1.36 ±0.01 ^d^
Isoleucine	0.76 ± 0.01 ^a^	0.79 ± 0.01 ^b^	0.81 ± 0.01 ^c^	0.76 ± 0.01 ^d^
Lysine	0.95 ± 0.01 ^a^	1.10 ± 0.01 ^b^	0.98 ± 0.01 ^c^	1.20 ± 0.01 ^d^
Valine	0.86 ± 0.01 ^a^	0.94 ± 0.01 ^b^	0.96 ± 0.01 ^c^	0.89 ± 0.01 ^d^
Threonine	0.79 ± 0.02 ^a^	0.92 ± 0.01 ^b^	1.04 ± 0.01 ^c^	0.83 ± 0.01 ^d^
Arginine	1.02 ± 0.01 ^a^	1.19 ± 0.01 ^b^	1.30 ± 0.01 ^c^	1.03 ± 0.01 ^d^
Phenylalanine	0.67 ± 0.01 ^a^	0.93 ± 0.01 ^b^	1.05 ± 0.01 ^c^	0.77 ± 0.01 ^d^
Tyrosine	0.43 ± 0.01 ^a^	0.53 ± 0.00 ^b^	0.57 ± 0.06 ^c^	0.45 ± 0.01 ^d^
Methionine	0.18 ± 0.01 ^a^	0.26 ± 0.01 ^b^	0.30 ± 0.01 ^c^	0.21 ± 0.01 ^d^
Histidine	0.40 ± 0.01 ^a^	0.50 ± 0.01 ^b^	0.57 ± 0.01 ^c^	0.43 ± 0.01 ^d^
Tryptophan	0.06 ± 0.01 ^a^	0.11 ± 0.01 ^b^	0.07 ± 0.00 ^bc^	0.09 ± 0.01 ^bd^
NEAA				
Alanine	0.85 ± 0.01 ^a^	0.94 ± 0.01 ^b^	0.87 ± 0.06 ^c^	0.97 ± 0.01 ^bd^
Serine	1.01 ± 0.01 ^a^	1.12 ± 0.01 ^b^	1.23 ± 0.01 ^c^	1.01 ± 0.01 ^d^
Glycine	1.15 ± 0.01 ^a^	1.32 ± 0.01 ^b^	1.29 ± 0.01 ^c^	1.19 ± 0.01 ^d^
Proline	1.01 ± 0.01 ^a^	1.05 ± 0.01 ^b^	1.03 ± 0.01 ^c^	1.03 ± 0.02 ^d^
Aspartic acid	1.25 ± 0.01 ^a^	1.50 ± 0.01 ^b^	1.40 ± 0.01 ^c^	1.56 ± 0.01 ^d^
Glutamic	2.15 ± 0.03 ^a^	2.88 ± 0.03 ^b^	2.59 ± 0.01 ^c^	3.01 ± 0.03 ^d^
Cystine	0.09 ± 0.01 ^a^	0.07 ± 0.01 ^b^	0.04 ± 0.01 ^c^	0.09 ± 0.01 ^ad^
∑EAA	7.56 ± 0.003 ^a^	8.70 ± 0.003 ^b^	9.03 ± 0.003 ^c^	8.04 ± 0.003 ^d^
∑NEAA	7.51 ± 0.008 ^a^	8.88 ± 0.007 ^b^	8.88 ± 0.004 ^c^	8.84 ± 0.008 ^d^
∑AA	15.07 ± 0.004 ^a^	17.58 ± 0.002 ^b^	17.91 ± 0.00 ^c^	16.88 ± 0.003 ^d^

Note: Numbers followed by different superscript letters in the same row are significantly different ( *P* < 0.05). Numbers with the same superscript letter in the same row show no significant difference ( *P* > 0.05).

* Values represent the means of triplicate samples.

The levels of free amino acids in the diets supplemented with different formulated products with a dosage of 150 ml/kg of feed are presented in Table 1. All types of amino acids in the diets of KP1, KP2, KP3, and KP4 were significantly different ( *P* < 0.05), except for tryptophan, and there was no significant difference ( *P* > 0.05) between KP2, KP3, and KP4. Among the essential amino acids, leucine and arginine were found in the highest amounts in the KP1, KP2, KP3, and KP4 diets. There was no significant difference ( *P* > 0.05) in the alanine content between KP2 and KP3 diets and the cystine level in KP1 and KP3 diets. Of the nonessential amino acids, glutamic and aspartic acid represented a significant portion of all four diets.

The present study found significant differences in the overall free essential and nonessential amino acid pools in the KP1, KP2, KP3, and KP4 diets ( Table 1). One-way ANOVA results exhibited a marginally significant interaction between experimental diets in terms of essential amino acids (F _(3,8)_ = 11.371, *P* = 0.003), nonessential amino acids (F _(3,8)_ = 0.407, *P* = 0.752), and overall amino acid pools (essential plus nonessential) (F _(3,8)_ = 7.355, *P* = 0.011). Duncan's Post-hoc test revealed that the free essential amino acids (9.03 ± 0.003%), nonessential amino acids (8.88 ± 0.004%), and overall amino acid pools (17.91%) were significantly higher ( *P* < 0.05) in feed supplemented with CP3 products, followed by CP2, CP4, and CP1 products ( Table 1).

### Proximate and amino acid profile of the whole body of giant gourami

Commercial feed combined with a new formulation product significantly affected the proximate carcass composition of juvenile giant gourami. One-way ANOVA results showed a marginal interaction among group treatments in the case of protein contents (F _(3,8)_ = 1.522, *P* = 0.282), fat (F _(3,8)_ = 5.663, *P* = 0.022), carbohydrates (F _(3,8)_ = 1.862, *P* = 0.214), and crude fibre (F _(3,8)_ = 1.445, *P* = 0.300). Duncan's Post-hoc test revealed that the protein content (28.85 ± 0.45%), fat (2.67 ± 0.04%), carbohydrates (1.97 ± 0.09%), and crude fibre (0.83 ± 0.02%) were significantly higher ( *P* < 0.05) in the KP3 diet than in the other treatments. Meanwhile, the carcass protein content of fish fed KP1, KP2, and KP4 was not significantly different ( *P* > 0.05) between treatments. For the energy total, KP3 was significantly higher ( *P* < 0.05) than the other treatments ( Table 2). However, the moisture content of the carcass did not show any significant variation among the KP1, KP2, KP3, and KP4 diets.

**Table 2.** Whole-body proximate and amino acid composition of giant gourami after a 90-day feeding trial. Mean ± SD *.

	KP1	KP2	KP3	KP4
	%, dry wet basis			
*Proximate composition*				
Dry matter	64.59 ± 0.16 ^a^	64.51 ± 0.34 ^a^	64.14 ± 0.33 ^a^	64.24 ± 0.12 ^a^
Crude protein	28.64 ± 0.28 ^a^	28.07 ± 0.79 ^ab^	28.85 ± 0.45 ^c^	28.66 ± 0.44 ^ad^
Crude fat	2.79 ± 0.03 ^a^	2.88 ± 0.02 ^b^	2.67 ± 0.04 ^c^	3.00 ± 0.02 ^d^
Carbohydrate	1.38 ± 0.01 ^a^	1.99 ± 0.06 ^b^	1.97 ± 0.09 ^c^	1.31 ± 0.02 ^d^
Crude fibre	0.97 ± 0.02 ^a^	0.68 ± 0.01 ^b^	0.83 ± 0.02 ^c^	0.95 ± 0.04 ^d^
Ash	1.63 ± 0.02 ^a^	1.70 ± 0.02 ^b^	1.54 ± 0.01 ^c^	2.11 ± 0.04 ^d^
Energy total (kg calorie/100 g)	144.77 ± 1.58 ^a^	155.48 ± 1.26 ^b^	157.90 ± 0.91 ^c^	149.60 ± 0.29 ^d^
*Amino acid composition*				
EAA				
Leucine	2.13 ± 0.01 ^a^	2.37 ± 0.01 ^b^	2.42 ± 0.01 ^c^	2.26 ± 0.01 ^d^
Isoleucine	1.13 ± 0.01 ^a^	1.25 ± 0.01 ^b^	1.38 ± 0.01 ^c^	1.19± 0.01 ^d^
Lysine	2.77 ± 0.01 ^a^	3.16 ± 0.02 ^b^	3.88 ± 0.01 ^c^	2.86 ± 0.01 ^d^
Valine	1.26 ± 0.01 ^a^	1.40 ± 0.01 ^b^	1.32 ± 0.01 ^c^	1.35 ± 0.01 ^d^
Threonine	1.38 ± 0.02 ^a^	1.49 ± 0.01 ^b^	1.43 ± 0.01 ^d^	1.48 ± 0.01 ^d^
Arginine	1.58 ± 0.01 ^a^	1.71 ± 0.01 ^b^	1.63 ± 0.01 ^c^	1.70 ± 0.01 ^d^
Phenylalanine	1.02 ± 0.01 ^a^	1.11 ± 0.01 ^b^	1.08 ± 0.01 ^c^	1.11 ± 0.01 ^d^
Tyrosine	0.80 ± 0.01 ^a^	0.84 ± 0.00 ^b^	0.83 ± 0.01 ^c^	0.85 ± 0.06 ^d^
Methionine	0.15 ± 0.01 ^a^	0.21 ± 0.01 ^b^	0.18 ± 0.01 ^c^	0.16 ± 0.01 ^d^
Histidine	0.55 ± 0.01 ^a^	0.56 ± 0.01 ^ab^	0.59 ± 0.01 ^c^	0.57 ± 0.01 ^d^
Tryptophan	0.08 ± 0.01 ^a^	1.02 ± 0.01 ^b^	1.08 ± 0.01 ^c^	0.06 ± 0.00 ^d^
NEAA				
Alanine	1.86 ± 0.01 ^a^	2.08 ± 0.01 ^b^	2.92± 0.01 ^c^	1.97 ± 0.01 ^d^
Serine	1.28 ± 0.01 ^a^	1.31 ± 0.01 ^b^	1.26 ± 0.01 ^c^	1.31 ± 0.01 ^d^
Glycine	1.58 ± 0.01 ^a^	1.68 ± 0.01 ^b^	1.61 ± 0.01 ^c^	1.77 ± 0.01 ^d^
Proline	1.06 ± 0.01 ^a^	1.16 ± 0.01 ^b^	1.08 ± 0.01 ^c^	1.16 ± 0.01 ^d^
Aspartic acid	2.71 ± 0.01 ^a^	3.08 ± 0.01 ^b^	3.79± 0.01 ^c^	2.77 ± 0.01 ^d^
Glutamic	4.36 ± 0.03 ^a^	4.92 ± 0.01 ^b^	4.97± 0.01 ^c^	4.66 ± 0.01 ^d^
Cystine	0.06 ± 0.01 ^a^	0.09 ± 0.01 ^b^	0.06 ± 0.01 ^c^	0.05 ± 0.01 ^d^
∑EAA	12.68 ± 0.003 ^a^	15.13 ± 0.005 ^b^	15.82± 0.001 ^c^	13.61 ± 0.008 ^d^
∑NEAA	12.91 ± 0.007 ^a^	14.32 ± 0.01 ^b^	15.69 ± 0.002 ^c^	13.50 ± 0.001 ^d^
∑AA	25.59 ± 0.003 ^a^	29.45 ± 0.04 ^b^	31.51 ± 0.001 ^c^	27.11 ± 0.004 ^d^

Note: Numbers followed by different superscript letters in the same row are significantly different ( *P* < 0.05). Numbers with the same superscript letter in the same row show no significant difference ( *P* > 0.05).

* Values represent the means of triplicate samples.

The mean quantities of total amino acids in the carcasses of *O. goramy* fed different diets are given in Table 2. Lysine and leucine represented a significant portion of the essential amino acids of the whole body carcass, and methionine was present in small quantities in all of the whole-body meat. Of the nonessential amino acids, glutamic acid, aspartic acid, and alanine were the highest, and cystine was the lowest for all whole-body carcasses of giant gourami fed different diets. The levels of glutamic acid were significantly higher in carcasses of fish fed the KP3 diet than in those provided the KP1, KP2, and KP4 diets.

When the overall quantities of total essential and nonessential amino acids were compared, the whole-body carcass amino acid content was significantly lower ( *P* < 0.05) in fish fed the KP1 diet than in those fed the KP2, KP3, and KP4 diets ( Table 2). The number of amino acids (essential plus nonessential) in the carcasses of fish fed the KP3 diet was significantly higher than that in fish fed the KP1, KP2, and KP4 diets.

### Growth coefficient and survival

**Figure 2.**
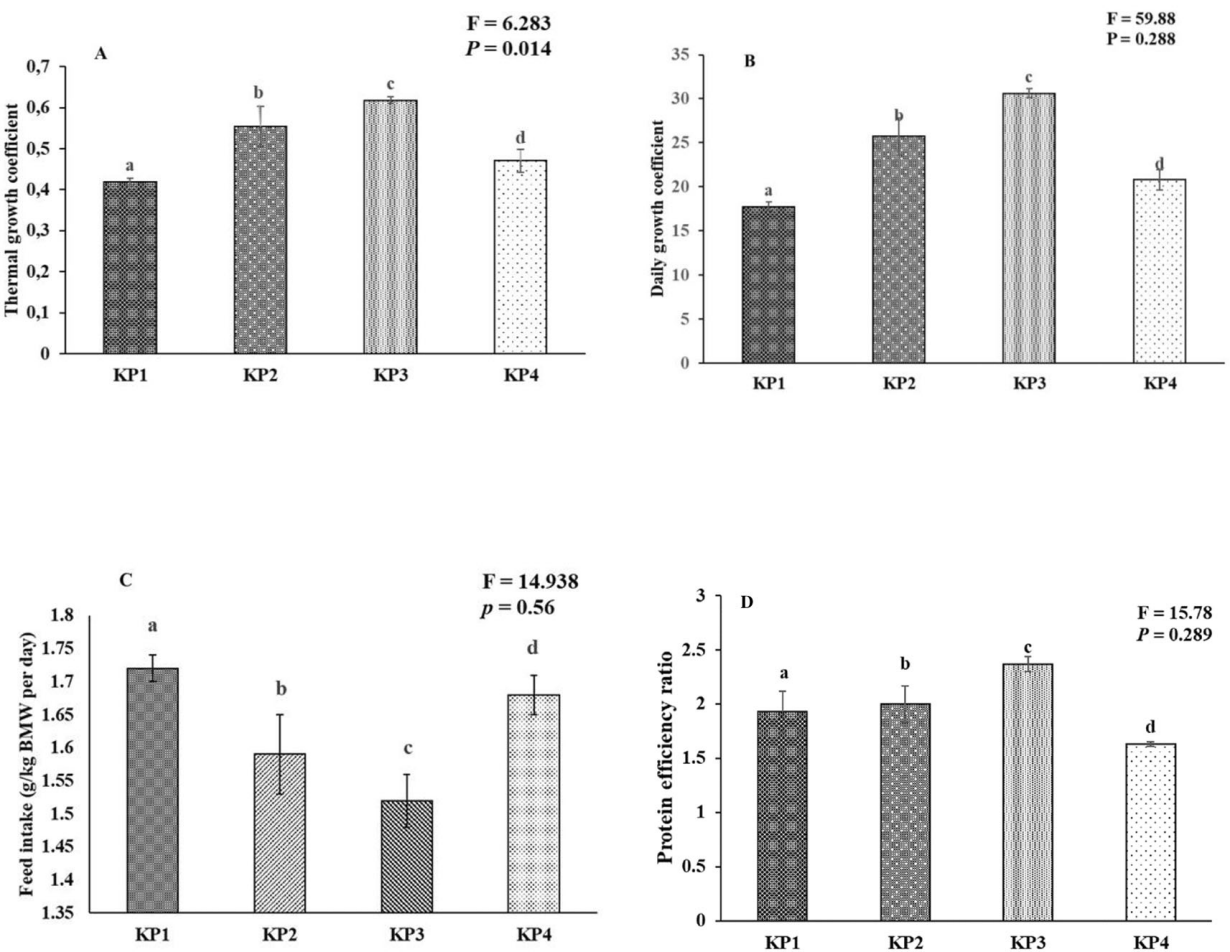
Growth coefficient and feed utilization of the giant gourami juveniles reared under different diets during 90 days of the experiment period. (A) Thermal growth coefficient (TGC), (B) daily growth coefficient (DGC), (C) feed intake (FI), and (D) protein efficiency ratio (PER). The mean value and standard deviation (mean ± SD) are presented for giant gourami (n = 3). Different superscripts in the bar diagram of the giant gourami juvenile TGC, DGC, FI, and PER indicate significant differences among other diets ( *P* < 0.05, One-way ANOVA Duncan Post-Hoc).

The growth coefficient and feed utilization of the giant gourami juveniles displayed significant differences among the diets. One-way ANOVA results exhibited a marginally significant difference between experimental diets in the case of the thermal unit growth coefficient (F _(3,8)_ = 153.99, *P* = 0.458), and daily growth coefficient (F _(3,8)_ = 59.88, *P* = 0.288), while total feed intake (% BW day-1) (F _(3,8)_ = 14.938, *P* = 0.56), and protein efficiency ratio (F _(3,8)_ = 15.78, *P* = 0.29) also showed significant differences ( *P* < 0.05) among the treatment diets ( Figure 2).

**Figure 3.**
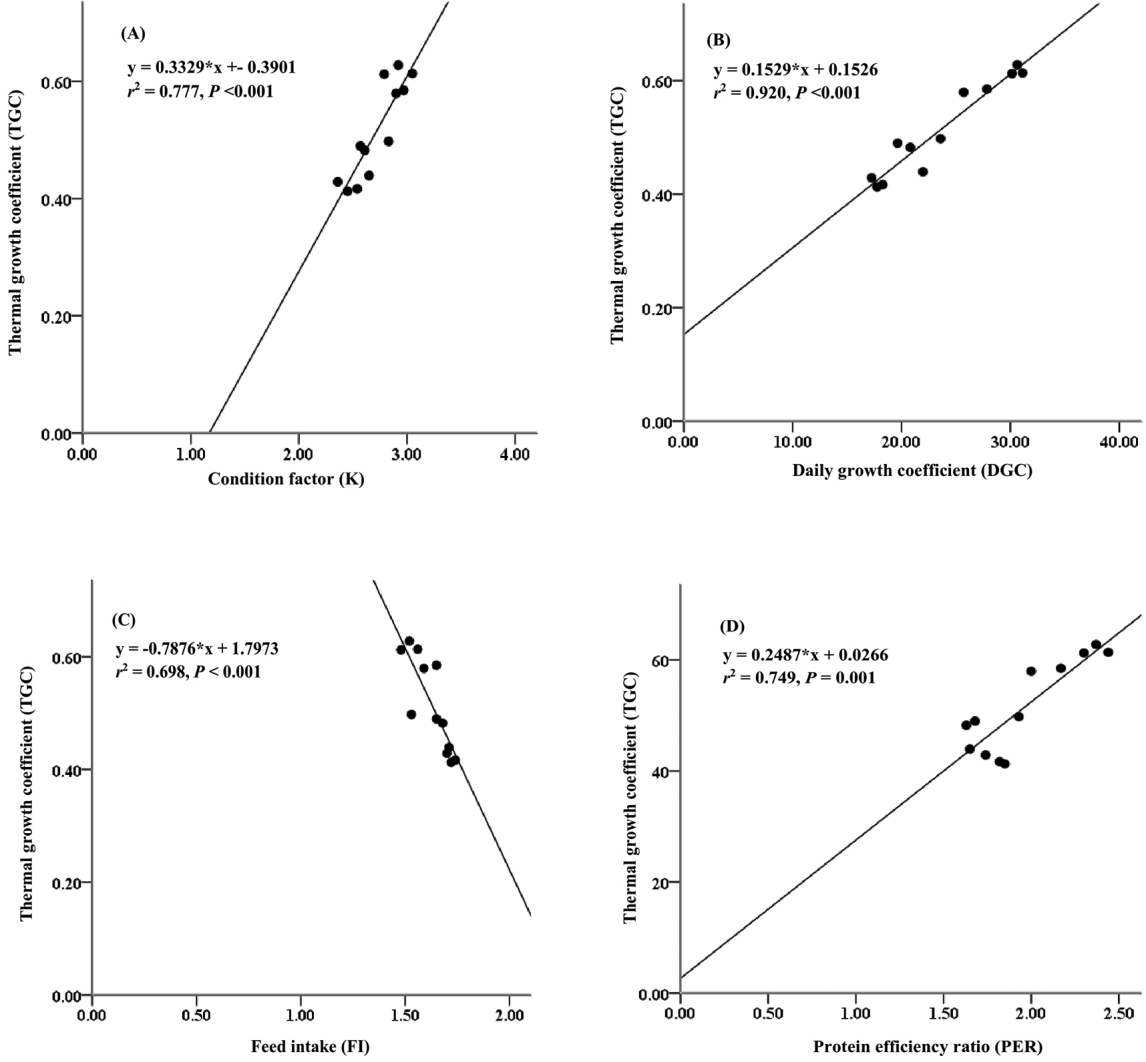
Relationships between thermal growth coefficient and condition factor (A), daily growth coefficient (B), feed intake (C) and protein efficiency ratio (D) for giant gourami ( *O.* *gourami*) over 90 days.

Furthermore, the thermal growth coefficient (TGC) has often been used to predict growth performance and production performance of aquaculture using water temperature at the fish-rearing location. This study presents the relationship between thermal growth coefficient and condition factor, daily growth coefficient, and protein efficiency ratio ( Figure 3). The thermal growth coefficient had strong relationships with the condition factor ( *r* ^2^ = 0.777, Figure 3A), daily growth coefficient ( *r* ^2^ = 0.920, Figure 3B), and protein efficiency ratio ( *r* ^2^ = 0.749, Figure 3D), while the thermal growth coefficient had a moderate relationship with the feed intake ( *r* ^2^ = 0.698, Figure 3C).

### Condition factor and body indices of giant gourami after 90 days of feeding

The condition factor was significantly different between diets (F _(3,8)_ = 19.98, *P* = 0.566) in the present study; while the GSI, HIS, and VFSI displayed marginally significant differences between diets. The HIS was significantly (F _(3,8)_ = 5.389, *P* = 0.500) higher in the KP3 diet, but KP1, KP2, and KP4 diets had no significant differences among them ( Table 3). The GSI value of giant gourami was significantly (F _(3,8)_ = 10.492, *P* = 0.243) different between diets, and the GSI of giant gourami fed KP3 rations was higher than if fed KP1, KP2, or KP4 diets. The VFSI was not considerably different among the KP1, KP2, and KP4 diets. The Duncan's post-hoc test revealed that the HIS (1.30 ± 0.13%), GSI (4.15 ± 0.36%), and VFSI (2.75 ± 0.34%) were significantly higher ( *P* < 0.05) in the KP3 diet than in the other diets. Meanwhile, BSI showed no significant difference ( *P* > 0.05) among the treatment diets ( Table 3).

**Table 3.** Mean (± SD) value condition factor and body indices of giant gourami during the 90-day experimental period.

Growth coefficients	KP1	KP2	KP3	KP4
Condition factor (CF)	2.45 ± 0.09 ^a^	2.90 ± 0.07 ^b^	2.92 ± 0.13 ^c^	2.61 ± 0.04 ^d^
Viscerosomatic index (GSI%)	3.20 ± 0.21 ^a^	3.77 ± 0.09 ^b^	4.15 ± 0.36 ^c^	3.17 ± 0.02 ^d^
Hepatosomatic (HIS%)	0.97 ± 0.05 ^a^	1.06 ± 0.19 ^ab^	1.30 ± 0.13 ^c^	1.04 ± 0.12 ^ad^
Visceral fat-somatic indexes (VFSI%)	2.15 ± 0.13 ^a^	2.29 ± 0.22 ^ab^	2.75 ± 0.34 ^c^	1.74 ± 0.21 ^ad^
Bilesomatic (BSI%)	10.11 ± 0.76	10.58 ± 1.01	10.48 ± 1.28	10.29 ± 0.77

Note: Numbers followed by different superscript letters in the same row are significantly different ( *P* < 0.05). Numbers with the same superscript letter in the same row show no significant difference ( *P* > 0.05).

### Gut micromorphology

**Figure 4.**
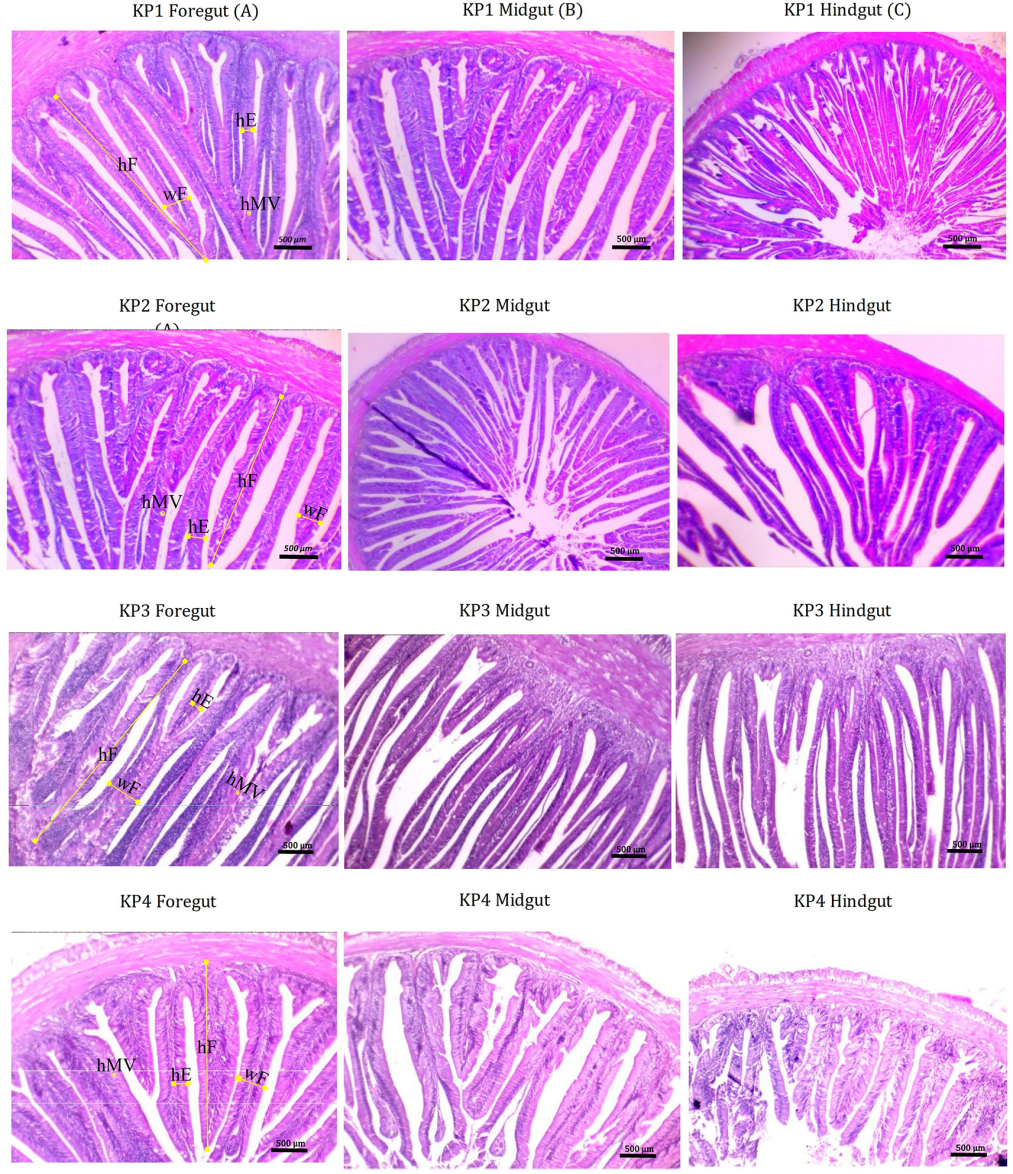
Light microscopy of the foregut (A), midgut (B) and hindgut (C) morphology of juvenile giant gorami of sago strain showing fold height (hF), enterocyte height (hE), and microvillus height (hMV) in fish fed KP1, KP2, KP3, and KP4 over 90 days. (Scale bar: 500μm; magnification ×100).

The gut morphometric measurements of giant gourami juveniles are presented in Table 4. Fish gut micromorphology was significantly affected by different feeds. One-way ANOVA results showed a significant effect of feed differences between groups in terms of foregut fold height (F _(3.8)_ = 816.70, *P* = 0.135), foregut fold width (F _(3.8)_ = 129.34, *P* = 0.974), height of the foregut (F _(3.8)_ = 169,80, *P* = 0.882), and microvillus height of the foregut (F _(3.8)_ = 56,01, *P* = 0.285). The Duncan's post-hoc test demonstrated that the foregut fold height (434.13 ± 1.76 μm), fold width (53.23 ± 0.88 μm), enterocyte height (27.42 ± 0.42 μm), and microvillus height (2.79 ± 0.45 μm) were significantly higher ( *P* < 0.05) in fish fed the KP3 diet than those fed the other diets. For the midgut, one-way ANOVA results showed a significant interaction among treatments in the case of fold height (F _(3,8)_ = 5602.628, *P* = 0.055), fold width (F _(3,8)_ = 129.341, *P* = 0.974), enterocyte height (F _(3,8)_ = 169.809, *P* = 0.882), and microvillus height (F _(3,8)_ = 56.016, *P* = 0.285). The Duncan's post-hoc test showed that the fold height of the midgut (324.96 ± 1.43 μm), fold width (61.50 ± 1.02 μm), and enterocytes (32.82 ± 0.54 μm) were significantly higher ( *P* < 0.05) in fish fed the KP3 diet, whereas microvillus height was significantly higher in fish fed the KP2 diet ( Table 4). Fish fed the KP3 diet showed a higher fold height of the hindgut (F _(3,8)_ = 5459.01, *P* = 0.066), fold width (F _(3,8)_ = 271.94, *P* = 0.865), enterocyte height (F _(3,8)_ = 299.180, *P* = 0.821), and microvillus height (F _(3,8)_ = 253.57, *P* = 0.316) ( Figure 4).

**Table 4.** Gut micromorphology of giant gourami juveniles fed different diets for 90 days.

	Foregut				Midgut				Hindgut			
	hF (μm) ^a^	wF (μm) ^b^	hE (μm) ^c^	hMV (μm) ^d^	hF (μm)	wF (μm)	hE (μm)	hMV (μm)	hF (μm)	wF (μm)	hE (μm)	hMV (μm)
KP1	336.17 ± 5.59 ^a^	51.30 ± 0.85 ^a^	26.21 ± 0.43 ^a^	2.56 ± 0.45 ^a^	227.50 ± 0.16 ^a^	47.16 ± 0.78 ^a^	24.31 ± 0.31 ^a^	1.64 ± 0.03 ^a^	213.92 ± 0.19 ^a^	42.91 ± 0.59 ^a^	20.22 ± 0.25 ^a^	1.49 ± 0.02 ^a^
KP2	343.43 ± 1.38 ^b^	52.14 ± 0.86 ^b^	26.84 ± 0.44 ^b^	2.77 ± 0.45 ^b^	274.61 ± 1.21 ^b^	58.12 ± 0.97 ^b^	29.87 ± 0.49 ^b^	1.85 ± 0.01 ^b^	243.51 ± 1.07 ^b^	53.01 ± 0.88 ^b^	28.00 ± 0.46 ^b^	1.64 ± 0.01 ^b^
KP3	434.13 ± 1.76 ^c^	53.23 ± 0.88 ^a^	27.42 ± 0.42 ^c^	2.79 ± 0.45 ^c^	324.96 ± 1.43 ^c^	61.50 ± 1.02 ^c^	32.82 ± 0.54 ^c^	1.80 ± 0.03 ^c^	305.60 ± 1.35 ^c^	60.02 ± 0.99 ^c^	29.54 ± 0.49 ^c^	1.77 ± 0.02 ^c^
KP4	321.18 ± 1.42 ^d^	50.20 ± 0.83 ^d^	25.62 ± 0.79 ^d^	2.31 ± 0.07 ^d^	228.45 ± 1.01 ^d^	56.95 ± 0.95 ^d^	29.19 ± 0.48 ^d^	1.69 ± 0.01 ^d^	217.69 ± 0.96 ^d^	61.64 ± 1.03 ^d^	24.32 ± 24.32 ^d^	1.40 ± 0.01 ^d^

Mean values with different superscript letters in the same line are significantly different ( *P* < 0.05).

^a^ hF = fold height.

^b^ wF = fold width.

^c^ hE = enterocyte height.

^d^ hMV = microvillus height.

### Pond water quality

The pond water quality values of the giant gourami juvenile rearing freshwater concrete pond were recorded; water temperatures, dissolved oxygen (DO), total alkalinity, hardness, pH, and nitrates were in the range of typical values as given by WHO/FAO, as shown in Table 5.

**Table 5.** The average values and range of water quality parameters in the concrete pond during the 90-days of experiment.

Water quality parameters	n	Mean ± SD	Range	WHO/FAO limits	References
Water temperatures (°C)	45	28.01 ± 1.06	27–30	25–33	Prokoso *et al.* ^43^
Dissolved oxygen (mg/L)	14	6.01 ± 0.14	5.80–6.20	3–5	Syandri *et al.* ^44^
Total alkalinity (mg/L as CaCO _3_)	14	58.09 ± 3.33	52.5–62.5	120	Boyd *et al.* ^45^
Hardness (mg/L as CaCO _3_)	14	66.34 ± 1.32	65–68.5	168	Boyd *et al.* ^45^
pH	14	7.48 ± 0.19	7.2–7.8	6.5–9.0	Boyd *et al.* ^45^
Nitrates (mg/L)	14	0.04 ± 0.01	0.03–0.05	0.2–219	Boyd and Tucker ^46^

## Discussion

The chemical analysis of fish feed is essential because it provides valuable information to aquafeed nutritionists concerned with readily available sources of proximate and amino acid compositions, including minerals and vitamins. This study investigated the nutritional quality of fish feed enriched with three different formulation products and one as a placebo. Dietary protein levels for giant gourami ranged from 19.68 to 21.70%. Overall, the crude protein content in the feed of this study was within the ranges observed by other authors. ^47^^–^^49^ The giant gourami belongs to the trophic level of herbivorous fish. ^50^ Generally, herbivorous fish require a lower dietary protein level than carnivorous fish. ^51^^,^^49^ Reducing the protein content of aquafeed is one method to increase continuous fish farming, by diminishing feed costs and reducing the impact on the aquatic environment. ^2^^,^^52^ The fat content of the feed ranged from 3.41 to 3.67%, which is similar to the feed fat content for juvenile grass carp, *Ctenopharyngodon idella*, ^53^ and lower than the feed fat content for the herbivorous fish *Ancistrus cirrhosis* ^48^ and for rearing rohu, *Labeo rohita*. ^54^ At the same time, the carbohydrate content of all feed treatments ranged from 26.37 to 31.19%, and the energy total (kg calorie/100 g) was between 234.41 and 240.87. Although protein content as an energy source for the maintenance and growth of giant gourami is relatively low, energy can be acquired from either protein or nonprotein sources, *i.e.*, fat and carbohydrates.

In the present study, the commercial fish feed was enriched with natural sources, *i.e.*, formulated products of mature coconut water and palm sap sugar fermented with various fungi ( *Aspergillus niger*, *Rhizopus oligosporus*, and *Saccharomyces cerevisiae*). In the recent past, the dose used was 300 ml/kg of feed. This method is a new approach that has been developed by Azrita *et al.* ^9^ to improve feed nutrition and whole-body carcasses, covering fatty acids, the atherogenic index and thrombogenic, feed efficiency, and growth performance of giant gourami. Here, we continued the investigation by reducing the feed dose to 150 ml/kg. This study's results found that supplementing feed with newly formulated products can increase feed nutrition, covering amino acids in diet and body meat, and the growth coefficient of giant gourami. Several authors have reported increasing feed nutrition and maximizing the digestive enzyme activity of aquacultured fish by providing feed supplemented with EPA and DHA, ^17^ iodine and selenium, ^10^ methionine, ^12^ fish oil, ^19^^,^^11^ and soybean oil. ^20^ In addition, the provision of feed has been supplemented with probiotics, ^21^ glycine, and prebiotics. ^22^ In this study, mature coconut water and palm sap sugar solution fermented with various fungi were used to supplement fish feed. In addition to coconut water and palm sugar, mushrooms also play a role in increasing feed nutrition. However, it's better to use *Rhizopus oligosporus.* As in the present study, Varzakas ^55^ and Vong *et al.* ^56^ showed that *Rhizopus oligosporus* can produce various extracellular enzymes. *Aspergillus niger.* has a high capacity to degrade antigenic proteins, including carbohydrases, proteases, lipases, and phosphatases, when used for fermenting plant-sourced fish feed ingredients. ^12^^,^^57^ *Saccharomyces cerevisiae* is one of the most acclaimed microorganisms. Its effectiveness is due to its useful composition, such as “β-glucans, nucleic acids, mannan oligosaccharides and chitin,” which are used for fermented ingredients. ^7^^,^^58^

The amino acid composition can be used to assess feed quality. Leucine, arginine, and glutamic acid were the most abundant free amino acids in the KP1, KP2, KP3, and KP4 diets. Similarly, in other studies on fish feed, such as feed for largemouth bass, *Micropterus salmoides*, the feeds were supplemented with glycine, prebiotics, and nucleotides in a soybean meal-based diet. ^22^ Feed for pacu, *Piaractus mesopotamicus,* was supplemented with an essential amino acid, ^59^ and feed for snubnose pompano, *Trachinotus blochii*, was supplemented with different levels of protein. ^60^ Apparently, supplementing feed with different ingredients is common, and in other species, leucine, arginine, and glutamic acid were the most abundant FAAs. Conversely, methionine levels were low in all experimental feeds. Methionine is one amino acid that must be available in fish feed because methionine is needed to protect body cells from stress. For optimal growth of juvenile hybrid grouper, 1.89% methionine is required in the feed. ^18^ The experimental feed contained 0.18–0.30% methionine, but whether this amount is sufficient for the needs of giant gourami is poorly understood.

In the current study, the nonessential amino acid compositions were slightly higher than the essential amino acid compositions in all the experimental diets. It was higher in the KP3 diet than the other diets. In contrast, the essential amino acids of fish feed for snubnose pompano were slightly higher than the nonessential amino acids content. ^60^ This difference may be caused by differences between freshwater fish and marine fish. As in the present study, Prabu *et al.* ^60^ reported that different dietary protein levels also caused different pools of FAAs, including limiting essential amino acid types in the diet ^59^ and supplemental glycine, prebiotic, and nucleotide levels in the soybean meal-based diet. ^22^ In the present study, this difference in FAA content is caused by various mushrooms used in the formulated products.

Giant gourami juveniles fed the KP3 diet showed higher levels of glutamic acid, aspartic acid, leucine, and lysine and lower levels of tyrosine, methionine, histidine, tryptophan, and cystine in their carcasses than those fed other diets. The carcasses of giant gourami fed the KP3 diet showed the highest sum of FAAs compared to cultured fish fed the KP1, KP2, and KP4. The differences in the FAA profile in the whole-body carcasses of giant gourami could be related to the fungus type used in the formulated products for enriched feed. Each type of mushroom has a different function depending on the fermented fish feed ingredients and is correlated with the whole-body carcass amino acids. ^12^^,^^57^ The FAA profile differences could be related to different aspects, such as diet composition, ^61^ dietary protein level, ^62^ and methionine levels in the diet, ^18^ including the water quality of the ponds. ^63^ This study does not analyse the relationship between growth performance and FAA profile or pond water quality. Several authors have reported that the physiological parameters of water quality and animal body composition are usually interrelated. ^64^^,^^60^ The present study did not examine whether the difference in FAAs in the whole-body carcass is correlated to pond water quality.

The lower weight gain of fish fed the KP1 diet compared to fish fed the KP2, KP3, and KP4 diets shows that a deficiency of either fungus in the formulated product for the enriched diet could lower the protein content and related sum amino acids, leading to the inhibition of giant gourami growth. In addition, it also affects feed intake and feed conversion ratios. The low protein efficiency ratio and daily growth coefficient in fish provided the insufficient KP1 diet were perhaps due to an amino acid imbalance. The amino acid content of the KP2, KP3, and KP4 diets increased, ranging from 16.88% to 17.91% after fermentation. The increase may be due in part to the increased protein content in the KP2, KP3, and KP4 diets, which was in line with the results of Jannatullah *et al.* ^57^ and Li *et al.*, ^12^ who found that *Aspergillus niger* and *Aspergillus awamori* fermentation increased the amino acid content of soybean meal by 2.56% and 15.56%, respectively. In addition, Dawood *et al.* ^36^ stated that the essential amino acid profile was changed after fermentation by *Saccharomyces cerevisiae.* This might result from the different fungi used having different utilization patterns for amino acids in this study. It influences the growth performance and nutrient utilization of giant gourami juveniles. We found that the methionine proportion was lower in the diets in the current study. In addition, methionine is an essential amino acid that plays a unique role in protein structure and metabolism. ^18^ It is possible that *Aspergillus niger*, *Rhizopus oligosporus*, and *Saccharomyces cerevisiae* fermentation promoted the conversion of specific amino acids to methionine. However, the exact mechanisms need to be studied further.

In the present study, the thermal growth coefficient (TGC) strongly correlated with the daily growth coefficient (DGC). Because faster daily fish growth requires a quality diet and constant water temperature during the rearing period, in this study, water temperature ranged from 27 to 30°C, and dissolved oxygen was between 5.8 and 6.2 mg/L. According to Besson *et al.*, ^65^ higher daily energy availability in the diet can lead to faster-growing fish, which is supported by constant water temperature and higher daily oxygen levels. The thermal growth coefficient had an essential change in environmental value. ^66^ Therefore, it was very important to keep the water temperature and dissolved oxygen constant in the aquaculture locations. At the same time, 78% of TGC values were determined by the condition factor connected to whole body weight and the total fish length. TGC of Atlantic cod, *Gadus morhua*, is influenced by body size and condition factors. ^67^

In this study, a higher value of TGC was detected in fish fed KP3; the effect is that the daily growth coefficient, and the protein efficiency ratio is better. Conversely, decreasing TGC has two effects, *i.e.*, a slow fish growth and lowered daily feed intake. Many scientists state that in aquaculture operations, net yield (kg/m ^3^) depends upon TGC fluctuation, feed intake, and daily oxygen consumption. ^65^^,^^68^^,^^69^

In the present study, food enrichment with different formulated products did not affect HIS or VFSI except in the KP3 diet. Whereas GSI is influenced by differences in diet, it did not affect BSI. The condition factor of largemouth bass, *Micropterus salmoides* (1.49–1.52%), fed enriched 1–2% EPA + DHA ^17^ was different from the value (0.68) reported by Arriaga-Hernandez *et al.* ^70^ for white snook ( *Centropomus viridis*) juveniles fed a 15% replacement of fish meal with soybean meal. Moreover, Hassan *et al.* ^71^ reported condition factor values ranging from 1.52 to 2.95 and an HSI between 1.4 and 1.5 for *Lates calcarifer* under different feeding rates (3–9% body weight d ^-1^). Barbosa *et al.* ^72^ reported VSI and LSI values of 2.24 and 3.86, respectively, for *Centropomus parallelus* fed a commercial diet. On the other hand, Syed *et al.* ^64^ also reported HSI and VSI values of 3.41 and 4.90, respectively, for *Oreochromis niloticus* at different levels of aloe vera extract as feed additives. In our study, the VSI of giant gourami ranged from 3.17 to 4.15, and the LSIs were between 1.74 and 2.75, both higher than those recorded at different stocking densities of giant gourami. ^44^ The high content of visceral fat observed in fish fed the KP3 diet might be explained by the diet having fat contents that exceed the needs of giant gourami juveniles and by the reduced energy expenditure of fish that are confined to rearing frame nets. Therefore, further analysis is necessary to determine the optimum dosage of the formulated product for the enrichment of feed to improve the growth performance of giant gourami.

For fish, the gut plays a significant role in absorbing nutrients, which is closely related to feed utilization. ^18^^,^^73^ Rossi *et al.* ^22^ demonstrated that the development of enterocytes affected the nutrient-absorbing efficiency of the gut of *Micropterus salmoides.* Feeding *Lates calcarifer* juveniles with the same basal diet supplemented with 1% probiotic yeast, *Saccharomyces cerevisiae*, and lactic acid bacteria, *Lactobacillus casei,* revealed a higher number of gut mucosal goblet cells and increased microvillous length. ^74^ In contrast, substituting as much as 12.5–25% soya protein concentrates with lupin ( *Lupinus albus*) meal in carp ( *Cyprinus carpio*) diets does not significantly affect the villi length and villi width of the gut. ^75^ In the current study, enriched feed with products supplemented from coconut water, palm sap sugar, and fungus significantly affected the micromorphology and gut size. The fold height, fold width, enterocyte height, and microvilli of fish fed the KP3 diet were higher than those of fish fed the KP1, KP2, and KP4 diets. The KP3 diet is a relevant formulated product to enrich commercial feed to promote the development of the gut in animal experiments, which may somewhat describe the significant growth performance and feed efficiency used in this study.

Furthermore, the micromorphology gut size of giant gourami is smaller than that of juvenile hybrid grouper, ^18^ turbot, *Scophthalmus maximus*, ^12^ largemouth bass, *Micropterus salmoides*, ^22^ and common carp, *Cyprinus carpio*. ^75^ The trophic food habits of fish may also affect the gut's hF, wF, hE, and hMV size because these habits are correlated with the digestibility coefficient. Under natural conditions, giant gourami is an herbivorous fish, while grouper, largemouth bass, and turbot are predatory fish, and common carp are omnivorous. Whether giving fish from different trophic levels the same diet affects the size of gut hF, wF, hE, and hMV is poorly understood.

## Conclusions

The present investigation observed that feed enriched with newly formulated products made from mature coconut water and palm sap sugar, and fermented with various mushrooms, given to fish in a dose of 150 ml/kg substantially affected the amino acid composition of the diet and whole-body carcass of giant gourami juveniles. It also affected the growth coefficient, feed utilization, body indices, and gut micromorphology size. The thermal growth coefficient had a strong relationship with the daily growth coefficient ( *r* ^2^ = 92%), condition factor ( *r* ^2^ = 77%), and protein efficiency ratio ( *r* ^2^ = 75%), while a moderate relationship with the feed intake ( *r* ^2^ = 69%). The CP3 formulation was optimal for feed quality, and the KP3 diet was optimal for body carcass, growth coefficient, body indices, and the ability of the intestines for feed absorption. Thus, our study also informs fish farmers about culturing good quality giant gourami and fulfilling nutrition requirements for food security.

## Data Availability

Figshare: Underlying data for ‘Effect of feed enriched by products formulated from coconut water, palm sap sugar, and mushroom on the chemical composition of feed and carcass, growth performance, body indices, and gut micromorphology of giant gourami, *Osphronemus goramy* (Lacepède, 1801), juveniles’. https://doi.org/10.6084/m9.figshare.20407647. ^76^ This project contains the following underlying data:
-
Table 1. Raw data of the experimental diets’ proximate composition -
Table 2. Raw data of amino acid of feed experimental -
Table 3. Raw data of whole body carcass proximate composition -
Table 4. Raw data of amino acid of whole-body carcass -
Table 5. Daily growth coefficient, feed utilization and body indices of giant gourami after 90 days of feeding. -
Table 6. Raw data gut micromorphology of giant gourami juveniles fed different diets for 90 days Data are available under the terms of the Creative Commons Attribution 4.0 International License (CC-BY 4.0).
