# Supplementary material for: Effect of feed enriched by products formulated from coconut water, palm sap sugar, and mushroom on the chemical composition of feed and carcass, growth performance, body indices, and gut micromorphology of giant gourami, Osphronemus goramy (Lacepède, 1801), juveniles
Source: F1000Res. 2023 Sep 11;12:140. Originally published 2023 Feb 7. [Version 2] doi: 10.12688/f1000research.124706.2 (PMC10562794; doi:10.12688/f1000research.124706.2)
Supplement: Peer review history

# Reviewer report: Reviewer response for version 2

**DOI:** 10.5256/f1000research.155597.r205339

**Published:** 2023-10-09

**Authors:** Fatimah Hashim, Nor Omaima Harun

**Affiliations:** 1 BIOSES Research Interest Group, Faculty of Science & Marine Environment, Universiti Malaysia Terengganu, Terengganu, Malaysia

Approved

Is the work clearly and accurately presented and does it cite the current literature?

Yes

If applicable, is the statistical analysis and its interpretation appropriate?

Partly

Are all the source data underlying the results available to ensure full reproducibility?

Yes

Is the study design appropriate and is the work technically sound?

Yes

Are the conclusions drawn adequately supported by the results?

Yes

Are sufficient details of methods and analysis provided to allow replication by others?

Yes

Reviewer Expertise:

Cytotoxicity, microscopy techniques, protozoology

We confirm that we have read this submission and believe that we have an appropriate level of expertise to confirm that it is of an acceptable scientific standard.

# Reviewer report: Reviewer response for version 2

**DOI:** 10.5256/f1000research.155597.r205340

**Published:** 2023-09-21

**Authors:** Qadar Hasani

**Affiliations:** 1 Program Studi Sumberdaya Akuatik, Fakultas Pertanian, Lampung University, Bandar Lampung, Lampung, Indonesia

The article has been revised properly. I approve this article for indexing.

Is the work clearly and accurately presented and does it cite the current literature?

Yes

If applicable, is the statistical analysis and its interpretation appropriate?

Yes

Are all the source data underlying the results available to ensure full reproducibility?

Partly

Is the study design appropriate and is the work technically sound?

Yes

Are the conclusions drawn adequately supported by the results?

Yes

Are sufficient details of methods and analysis provided to allow replication by others?

Yes

Reviewer Expertise:

Plant Botany and Ecology, Pollution, and Environmental Chemistry, The Dynamics of the Plankton Community, fish biology, and fish nutrition.

I confirm that I have read this submission and believe that I have an appropriate level of expertise to confirm that it is of an acceptable scientific standard.

# Reviewer report: Reviewer response for version 1

**DOI:** 10.5256/f1000research.136930.r196596

**Published:** 2023-08-25

**Authors:** Qadar Hasani

**Affiliations:** 1 Program Studi Sumberdaya Akuatik, Fakultas Pertanian, Lampung University, Bandar Lampung, Lampung, Indonesia

**Abstract**

- **Background**: Please add the sentence "of juvenile giant gourami" to the end of the sentence.
- **Methods:** Scientific names of *Aspergillus niger*, *Rhizopus oligosporus*, and *Saccharomyces cerevisiae* "please write in italic"
- **Results:** Please add include the regression values (r ^2^) for the condition factor, feed intake, and protein efficiency ratio.
- **Conclusion:** Please add a sentence for the condition factor and body indices to the end of the sentence.

**Introduction**

- Please include the scientific name of the Azolla plant.
- Please maintain consistency with the term "whole-body carcass".
- Please enhance the third paragraph by introducing or used fermented mushrooms (specifically *Aspergillus niger*, *Rhizopus oligosporus*, and *Saccharomyces cerevisiae*).
- Please incorporate into the seventh paragraph the second objective of the research, which focuses on analyzing the condition factor and feed intake of giant gourami juveniles, along with their correlation to thermal growth coefficient, body index, and gut micromorphology.

**Methods: **

- Ethical approval part; Please explain, namely "Gurami sago" whether it is a new species or strain.
- Experimental procedures part and sampling; Please ensure consistency with the term "gurami sago." or changed to "giant gourami"
- Please add state the manufacturing country of the light microscope (Olympus IX71) equipped with Image-Pro Plus 7.0 software.

**Results:**

- The authors have displayed data concerning intestinal micromorphology (Table 4). It is recommended that the authors present all morphological findings related to intestinal micromorphology in the form of images/figures.

**Discussion: **

- Please be consistent with the name used for example giant gourami.

**Conclusions:**

- Please include regression values for each parameter, such as the condition factor (r ^2^ = 0.77) and protein efficiency ratio (r ^2^ = 0.74).
- Please maintain consistency with the term "whole-body carcass".

Is the work clearly and accurately presented and does it cite the current literature?

Yes

If applicable, is the statistical analysis and its interpretation appropriate?

Yes

Are all the source data underlying the results available to ensure full reproducibility?

Partly

Is the study design appropriate and is the work technically sound?

Yes

Are the conclusions drawn adequately supported by the results?

Yes

Are sufficient details of methods and analysis provided to allow replication by others?

Yes

Reviewer Expertise:

Plant Botany and Ecology, Pollution, and Environmental Chemistry, The Dynamics of the Plankton Community, fish biology, and fish nutrition.

I confirm that I have read this submission and believe that I have an appropriate level of expertise to confirm that it is of an acceptable scientific standard.

# Response

**Published:** 2023-08-26

**Authors:** Azrita Azrita

**Affiliations:** Universitas Bung Hatta, Indonesia

Reviewer Report response by Qadar Hasani, Aquatic Resources Study Program, Faculty of Agriculture, Lampung University, Bandar Lampung, Lampung, Indonesia.

**Abstract**

1. Background: We agree to add the sentence "of juvenile giant gourami" at the end of the sentence. These suggestions will be fixed in a new revised version.
2. Methods: We have agreed that some scientific names should be italicized as shown in the abstract, for example Aspergillus niger, Rhizopus oligosporus, and Saccharomyces cerevisiae. These suggestions will be fixed in a new revised version.
3. Results: We will add the regression value (r ^2^) for each parameter if the abstract does not exceed 300 words.
4. Conclusion: We will add the condition factor and body indices if the abstract does not exceed 300 words.

**Introduction**

1. We will add scientific name of Azolla plant (Azolla pinnata) in new revised version.
2. In the new revised version, we will consistently use the phrase "whole-body carcass"
3. We have mutually decided to include the scientific names of three specific species: Aspergillus niger, Rhizopus oligosporus, and Saccharomyces cerevisiae, in the upcoming revised version.
4. The seventh paragraph of the new revised version will encompass the second research objective. This objective entails investigating the condition factor concerning the feeding of giant gourami fingerlings and its correlation with the thermal growth coefficient, body index, and gut micromorphology.

**Methods**

1. Ethical Approval Section: The Sago gourami represents one of the giant gourami strains, and we will include it in the new revised version.
2. We will consistently write "Giant gourami" in the revised version of the article.
3. We will append a newly revised version of the article with the information that Japan is the nation where light microscopes (Olympus IX71) equipped with Image-Pro Plus 7.0 software are manufactured.
4. In order to enhance the data presented in Table 4, we will incorporate images or figures depicting intestinal micromorphology within the new version of the revision.

**Discussion**

1. In the revised version, we will consistently use the name "Giant gourami".

**Conclusions:**

1. In the new revised version, we agreed to include the coefficient of determination (r ^2^) in the conclusion section, for the condition factor (r ^2^ = 0.77) and the protein efficiency ratio (r ^2^ = 0.74).

# Reviewer report: Reviewer response for version 1

**DOI:** 10.5256/f1000research.136930.r163500

**Published:** 2023-02-28

**Authors:** Fatimah Hashim, Nor Omaima Harun

**Affiliations:** 1 BIOSES Research Interest Group, Faculty of Science & Marine Environment, Universiti Malaysia Terengganu, Terengganu, Malaysia

Dear authors, thank you for your paper and we appreciate the effort and contribution that you have made to the field.

 However, we noticed that your paper lacks results for the micromorphology part, which makes it difficult to fully understand the conclusions of your study. Specifically, we're unable to fully assess the morphological characteristics of the samples and how they relate to the overall findings. Therefore, we suggest that you consider adding some additional data/results where necessary. Additionally:

1. Some scientific names were not italicised.
2. The number at the beginning of the sentence must be spelt out.
3. Some literature in the introduction compares the use of coconut water on terrestrial animal or human levels. It is better to compare with aquatic organisms that are more related to the gourami.
4. The author should provide all morphology results on gut micromorphology (in the form of images/ figures), not only the data measurement (Table 4.) 

Additional comments from Fatimah Hashim attached here.

 Additional comments from Nor Omaima Harun attached here.

Is the work clearly and accurately presented and does it cite the current literature?

Yes

If applicable, is the statistical analysis and its interpretation appropriate?

Partly

Are all the source data underlying the results available to ensure full reproducibility?

Yes

Is the study design appropriate and is the work technically sound?

Yes

Are the conclusions drawn adequately supported by the results?

Yes

Are sufficient details of methods and analysis provided to allow replication by others?

Yes

Reviewer Expertise:

Cytotoxicity, microscopy techniques, protozoology

We confirm that we have read this submission and believe that we have an appropriate level of expertise to confirm that it is of an acceptable scientific standard, however we have significant reservations, as outlined above.

# Response

**Published:** 2023-04-16

**Authors:** Azrita Azrita

**Affiliations:** Universitas Bung Hatta, Indonesia

Reviewer Report by Fatimah Hashim, Universiti Malaysia Terengganu, Terengganu, Malaysia

 Nor Omaima Harun, Universiti Malaysia Terengganu, Terengganu, Malaysia

1. Author Respond: We agree that several scientific names should be italicized as in the Abstract *: Aspergillus niger, Rhizopus oligosporus, and Saccharomyces cerevisiae.*
2. Author response: We agree that when a number appears at the beginning of a sentence, the number must be spelled out with letters, just like the example in the Abstract where the number 100 is changed to One hundred, the number 2 liters is changed to Two litres, and 1 litres changed to One litres.
3. Author's Response: Previously, we stated in the introduction that animals or humans have used coconut water on land. However, no literature has been found testing the use of coconut water on aquatic animals, including gourami. Therefore, this study focuses on the novelty of using new product formulas for coconut water, palm sugar, and mushroom in aquatic animals, especially giant gourami.
4. Author response: Olympus Model IX71, HBO 100W, Phase Contrast Microscopy, Fluorite Lenses, Inverted Cell Culture, Fluorescence Microscope, Country: Japan.
5. Author response: We have prepared gut micromorphology images and are willing to display Figure 4 in the article if the editorial team approves the addition (Please open this link).
